# Glycation Leads to Increased Polysialylation and Promotes the Metastatic Potential of Neuroblastoma Cells

**DOI:** 10.3390/cells9040868

**Published:** 2020-04-02

**Authors:** Maximilian Scheer, Kaya Bork, Frieder Simon, Manimozhi Nagasundaram, Rüdiger Horstkorte, Vinayaga Srinivasan Gnanapragassam

**Affiliations:** 1 Institute for Physiological Chemistry, Medical Faculty, Martin-Luther-University Halle-Wittenberg, 06114 Halle (Saale), Germany; Maximilian.Scheer@uk-halle.de (M.S.); kaya.bork@medizin.uni-halle.de (K.B.); frieder.simon@uk-halle.de (F.S.); manimozhi.nagasundaram@medizin.uni-halle.de (M.N.); vinayaga.gnanapragassam@medizin.uni-halle.de (V.S.G.); 2 Department for Neurosurgery, University Hospital Halle, 06120 Halle (Saale), Germany

**Keywords:** (poly) sialic acid, NCAM, adhesion, migration, methylglyoxal, Kelly cells

## Abstract

Neuroblastoma is the second most frequent extracranial tumor, affecting young children worldwide. One hallmark of tumors such as neuroblastomas, is the expression of polysialic acid, which interferes with adhesion and may promote invasion and metastasis. Since tumor cells use glycolysis for energy production, they thereby produce as side product methylglyoxal (MGO), which reacts with proteins to advanced glycation end products in a mechanism called glycation. Here we analyzed the expression of (poly) sialic acid and adhesion of Kelly neuroblastoma cells after glycation with MGO. We found that both sialylation and polysialylation is increased after glycation. Furthermore, glycated Kelly neuroblastoma cells had a much higher potential for migration and invasion compared with non-glycated cells.

## 1. Introduction

Neuroblastoma is a malignant embryonic tumor and arises from immature cells of the sympathetic nervous system. Neuroblastomas represent neuroendocrine tumors because they synthesize catecholamines. They are among the most common extracranial solid tumor forms in childhood. Neuroblastoma is responsible for approximately 7.3% of childhood tumors and the cause of death in around 15% of childhood cancer. The mean age of onset of the disease is 14 months, whereas adults are rarely affected [1].

Characterization of genetic alterations in neuroblastoma revealed that MYCN amplification is an independent prognostic factor for identifying rapid tumor progression and predicting a poor prognosis irrespective of age and clinical stage [2]. The clinical picture depends also on the location of the tumor. In most cases, the primary tumors are located in the area of the adrenal gland, paravertebral or in the abdominal midline. About 50% of all patients already have distant metastases when diagnosed. These mostly affect the bone marrow (86% of all patients with metastatic disease), the bone (62%), the lymph nodes (19%) and the liver (17%) [1]. Neuroblastoma is a sporadic disease; family cases make up only about 1% of all patients. In a large part of these familial neuroblastomas, germline mutations of the ALK (anaplastic lymphoma kinase) gene can be detected [3]. Despite this frequent metastasis, spontaneous remission of the tumor including metastases has often been described. Spontaneous remissions occur in 50% of cases of stage 2a—and even in 80% of all cases in the rare stage 4 S [4,5,6]. As many tumors, also neuroblastomas express polysialylated neural cell adhesion molecule (NCAM) [7]. Polysialylation is a posttranslational modification of NCAM, in which long linear polymers of sialic acid are built [8]. Both, polysialic acid and NCAM expression are prognostic markers for neuroblastoma [9,10]. In addition to its role in cell migration and axonal growth during development, polysialic acid is closely related to tumor malignancy. The level of polysialic acid correlates with the malignant potential of several tumors, such as undifferentiated neuroblastoma and is significantly more abundant in high-grade tumors than in low-grade tumors [7]. It has been shown that also in aggressive brain tumors, such as glioblastoma, polysialylation is a negative prognostic marker, since the expression of long chains of negatively charged sialic acids interferes with cell adhesion and promotes metastasis [11,12,13].

Nearly all tumors including neuroblastoma use primarily glycolysis to generate energy [14]. Besides ATP and NADH, glycolysis produces dicarbonyl compounds that are highly reactive and react with amino groups [15]. One of the most common byproducts of glycolysis is methylglyoxal (MGO) [16]. Approximately 0.1–0.4% of the glucose is converted to MGO during glycolysis. MGO is 20,000 times more reactive than glucose reacting with amino groups [17]. This non-enzymatic chemical reaction of MGO with amino groups is also called glycation and stable advanced glycation end products (AGEs) are formed in further steps. AGEs represent a heterogeneous group of often unknown structures. However, some of these structures are well characterized, such as carboxymethyllysine (CML) and carboxylethyllysine (CEL), which are formed by glyoxal or MGO [18]. Those and other AGEs interfere with protein function, solubility and degradation [19]. The dicarbonyl compound MGO occurs in high concentrations in the human cerebrospinal fluid [20]. In this study, we analyzed Kelly neuroblastoma cells, which express both NCAM and polysialic acid [7] before and after glycation with MGO. We found an upregulation of polysialylation and in agreement with this observation, reduced cellular adhesion and increased invasion.

## 2. Materials and Methods

### 2.1. Chemicals and Reagents

Cell culture medium Dulbecco’s Modified Eagle Medium, serum, penicillin and streptomycin were purchased from Gibco (Darmstadt, Germany). Laminin, fibronectin and vitronectin were purchased from Sigma Aldrich (Hamburg, Germany). E- and CIM-plates were purchased from OLS (Bremen, Germany). Protease and phosphatase inhibitors were purchased from Sigma Aldrich (Hamburg, Germany). Biotinylated Maackia Amurensis Lectin II, Sambucus nigra lectin and DyLight 488 Streptavidin (Vector labs, Burlingame, CA, USA). Beta tubulin anti mouse antibody were purchased from Thermofisher (Darmstadt, Germany).

### 2.2. Cell Culture

Kelly cells were cultured in RPMI medium containing 10% FCS with L-glutamine, minimum essential medium, non-essential amino acids, penicillin and streptomycin. Cultured cells were treated with 0, 5, 10, 25, 50 and 100 µM methylglyoxal dissolved in medium. After 48 h, cells were washed two times with PBS and dissociated with PBS/EDTA. Cells were further washed with PBS and pelleted by centrifugation at 1000 rpm (82× *g*) for 5 min for further analysis.

### 2.3. Cell Viability Assay by MTT

Kelly cells (10,000/well) were seeded in 96-well plates and treated with MGO at various concentrations (0–100 µM) for 48 h. Cultured cells were replaced with fresh medium for every 24 h. After 48 h 200 µL of fresh medium were replaced, to that 20 µL of 5 mg/mL of 3-[4,5-Dimethylthiazol-2-yl]-2,5 diphenyl tetrazolium bromide (MTT) reagent was added and incubated for 4 h at 37 °C. After removal of the media, 150 µL of DMSO was added to each well. The plates were kept in a plate shaker for 20 min for solubilization of the formazan crystals followed by measuring the absorbance at 560 nm in the ELISA plate reader (Thermo Scientific Multiskan EX, Langenselbold, Germany).

### 2.4. HPLC Analysis of Sialic Acid

Kelly cells were incubated with MGO for 48 h, followed by PBS washing and dissociation using PBS/EDTA buffer. Cells were pelleted by centrifugation and further processed for sialic acid analysis. Briefly cell pellets were homogenized and followed by mild acid hydrolysis of Sia with an equal volume of 2 M propionic acid at 80 °C, for 4 h. After hydrolysis the total lysate was cooled on ice for 10 min, followed by centrifugation at 16,000× *g* for 20 min at 4 °C. The supernatant was subjected to centrifugation using Amicon^®^Ultra-0.5 centrifugal filter devices at 16,000× *g* at 4 °C, for 30 min. The flow through was collected, freeze-dried and lyophilized overnight to remove the acid. The samples were dissolved in 100 µL of water to which an equal volume of 1,2-diamino-4,5-methylenedioxybenzene (DMB) was added and incubated in the dark at 50 °C, for 2.5 h. Samples were cooled briefly on ice followed by short centrifugation at 10,600× *g* for 1 min. Then, 20 µL of DMB labeled samples were injected into the HPLC column and eluted the sialic acids using isocratic solvent, acetonitrile:methanol:water at 8:6:86 ratios at a flow rate of 0.6 mL/min. Neu5Ac standard was labeled with DMB and used as a reference.

### 2.5. Immunoblotting

Kelly cells were seeded in the 6-well plates and cultured for 48 h with the respective concentrations of MGO. Fresh medium containing MGO was replaced every 24 h. After 48 h, cells were washed with PBS and dissociated with PBS/EDTA. Cells were washed once again with PBS and pelleted by centrifugation. Cell pellets were lysed and solubilized with RIPA buffer containing protease and phosphatase inhibitors. A total of 50 μg of protein was loaded on the 10% SDS PAGE gels and separated at 80 V for 3 h. Resolved proteins were transferred onto nitrocellulose membranes and blocked overnight with TBS containing 5% milk for 1 h at room temperature. Blots were incubated with anti-polySia (mab 735; 1:1000); anti-NCAM (mab 123C3; 1:1000); anti-RAGE (mab ab3611; 1:1000); anti-CML-AGE (mab CML56; 1:10,000); anti-tubulin (mab BT7R; 1:5000) overnight at 4 °C. Blots were washed 3 times with TBS-Tween (TBST) and incubated with HRP conjugated anti-mouse secondary antibody at 1:10,000 dilution in TBST containing 3% milk for 1 h at RT. Again, blots were further washed 3 times with TBST for 10 min each. PolySia bands were developed using chemiluminescence reagent (Immobilon Forte Western HRP substrate: Merck, Darmstadt, Germany) and detected by ChemiDoc XRS system (Bio-Rad Laboratories GmbH, München, Germany).

### 2.6. Adhesion Assay

E-plates were coated with fibronectin, laminin or vitronectin, respectively at 20 µg/mL concentration and incubated for 60 min at 37 °C. The wells were blocked with 0.1% BSA for 1 h at 37 °C. The wells were washed with PBS and 0.1 × 10^6^ cells from MGO treatment were added to the respective E plate wells and allowed to settle the cells. Afterwards the E-plate was kept in the xCELLigence device (RTCA, OLS xCELLigence, Bremen, Germany) and the adhesion was quantified by monitoring the impedance for each 5 min for 4 h.

### 2.7. Migration Assay

The migration assay was performed in a real-time cell analyzer (RTCA, OLS xCELLigence, Bremen, Germany). Cells were treated with MGO for 48 h, washed twice with 10 mL of PBS and dislodged with PBS/EDTA buffer and washed once with PBS. Cells (0.5 × 10^6^/well) were added to the upper chamber of the 16-well CIM plate (OLS xCELLigence, Bremen, Germany). The lower chamber was previously filled with 160 µL of complete medium. After the cells were settled in the upper plate the CIM plate was placed in the station. The impedance was measured for every 15 min up to 24 h for monitoring the migration of the cells.

### 2.8. Invasion Assay

Kelly cells were treated with MGO for 48 h, washed twice with PBS and dislodged with PBS/EDTA buffer and washed once with serum-free medium. Cells were (0.5 × 10^6^/well) added to the upper chamber of the 16-well CIM-plate previously coated ECM gel (Engelbrecht-Holm-Swarm murine sarcoma) at 1:50 dilution. The lower chamber had been previously filled with 160 µL of complete medium. After the cells were settled in the upper plate (kept in the cell culture hood for 30 min), the CIM-plate was placed in the station (RTCA, OLS xCELLigence, Bremen, Germany). Invasion of the cells was monitored, by measuring the impedance for every 15 min up to 24 h.

## 3. Results

### 3.1. Glycation of Neuroblastoma Cells

**Figure 1:**
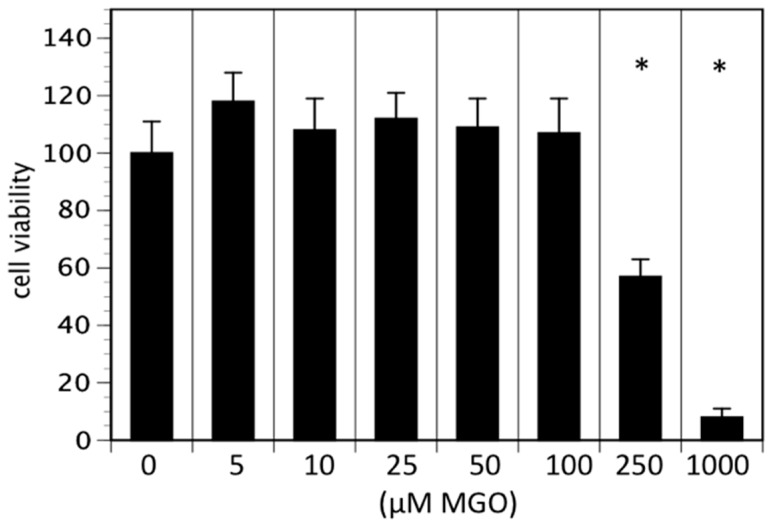
Cell viability in the presence of MGO. Kelly cells were grown in the absence or presence of various MGO concentrations for 48 h and an 3-[4,5-Dimethylthiazol-2-yl]-2,5 diphenyl tetrazolium bromide (MTT) assay was performed. Cells without MGO treatment were normalized to 100%. Bars represent means of +/− SEM of 3 independent experiments (* *p* < 0.05).

**Figure 2:**
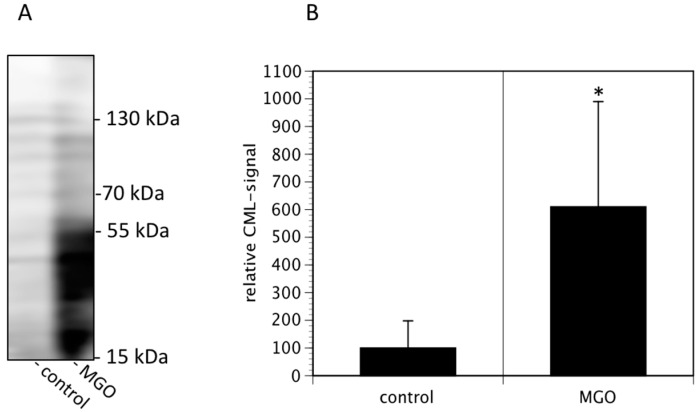
MGO treatment of Kelly cells leads to increased glycation. Kelly cells were grown in the absence or presence of 100 µM MGO for 48 h. Cells were washed and proteins were isolated, separated by SDS-PAGE and stained using an anti-CML-AGE antibody (**A**). The entire membrane is shown. (**B**) Bands of 3 independent experiments were scanned and the intensities analyzed by Image J. Bars represent means +/− SEM of 3 independent experiments (* *p* < 0.05).

In the first series of experiments, we analyzed the effect of MGO on the viability of neuroblastoma cells. We decided to use Kelly cells, since these cells are known to express both NCAM and polysialic acids [7]. We therefore investigated whether glycation was affecting the metabolic activity of the Kelly cells. An MTT assay was performed after growing Kelly cells in the absence or presence MGO for 48 h. Various concentrations up to 100 µM MGO did not significantly alter the metabolic activity of Kelly cells (Figure 1). However, at 250 µM MGO, the metabolic activity was reduced by about 50%, whereas treatment of Kelly cells with 1 mM MGO for 48 h was toxic for the cells (Figure 1). Therefore, we decided to use 100 µM MGO for all further experiments—a concentration that can also be reached in the cerebrospinal fluid of diabetic patients. Next, we analyzed whether treatment of Kelly cells leads to glycation or AGE formation. For this, Kelly cells were grown in the presence of 100 µM MGO for 48 h and analyzed by Western blot using a specific AGE-antibody. The blot indicated that treatment of Kelly cells with 100 µM MGO leads to increased AGE signals on the entire membrane (Figure 2A) Quantification reveals a 5-fold increase of the overall signal intensity (Figure 2B).

### 3.2. Glycation Leads to Increased Sialylation and Increased Expression of Polysialic Acids

**Figure 3:**
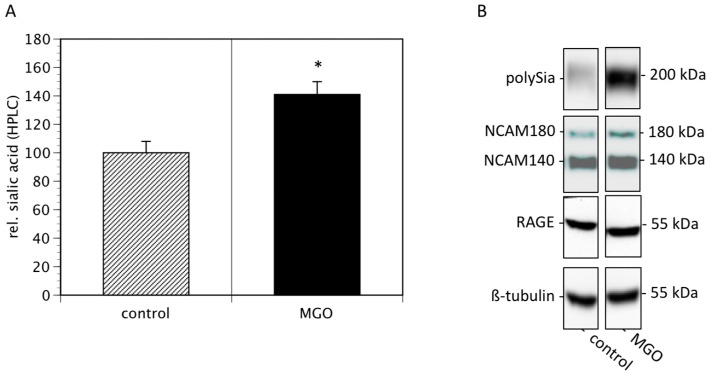
MGO treatment of Kelly cells leads to increased (poly)sialylation. Kelly cells were grown in the absence or presence of 100 µM MGO for 48 h. Cells were washed and proteins were isolated. (**A**) Proteins were dried, sialic acids were cleaved, labeled with DMB and analyzed by HPLC. Bars represent means +/− SEM of 3 independent experiments (* *p* < 0.05). (**B**). Proteins were separated by SDS-PAGE and transferred to a nitrocellulose membrane. Polysialylation was detected using monoclonal 735 antibody. NCAM expression was analyzed using the monoclonal 123C3 antibody. For control, RAGE and ß-tubulin staining was used.

Since cell surface sialylation and polysialylation are prognostic markers of several tumors [21], we then quantified membrane bound sialic acids and polysialic acids of Kelly cells cultured in the absence or presence of 100 µM MGO. We found that MGO treatment increased the total sialic acids on cell surface proteins by nearly 50% (Figure 3A). Analysis of polysialic acid expression revealed a dramatic increase in polysialylation after MGO treatment (Figure 3B, topmost bands). This was not due to increased NCAM expression, since the NCAM expression was not increased after MGO treatment (Figure 3B). For control reasons, we also determined the RAGE and tubulin expression, which are also not altered (Figure 3B).

### 3.3. MGO-Induced Polysialylation Interferes with Adhesion

**Figure 4:**
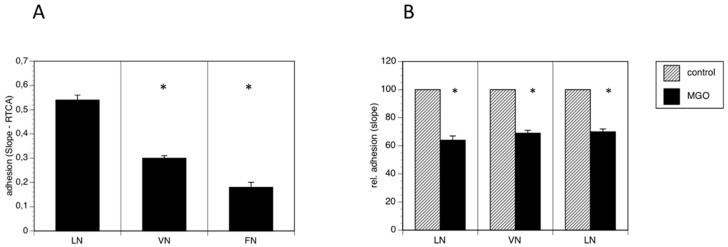
MGO-induced polysialylation interferes with adhesion. A total of 0.1 × 10^6^ Kelly cells were allowed bind to E-plates coated with laminin (LN), vitronectin (VN) or fibronectin (FN). (**A**) Adhesion was continuously quantified by RTCA measurement. Bars represent means +/− SEM of 3 independent experiments (* *p* < 0.05). (**B**) Kelly cells were grown in the absence (control) or presence of 100 µM MGO for 48 h prior to the adhesion assay. Adhesion in the absence of MGO was set to 100% (control) and in the presence of MGO in percent of the control. Bars represent means +/− SEM of 3 independent experiments (* *p* < 0.05).

Because polysialylation is often involved in the regulation of adhesion, we performed real time cell adhesion assays with Kelly cells on several substrates cultured in the absence or presence of MGO. First, we compared several substrates and found, that Kelly cells prefer laminin as substrate over vitronectin and fibronectin (Figure 4A). We then compared adhesion of Kelly cells to all three substrates grown presence of 100 µM MGO for 48 h. We measured a reduction of adhesion of about 30% on all tested substrates (laminin, vitronectin or fibronectin) (Figure 4B). Please note that we normalized all the data for comparison.

### 3.4. MGO-Induced Polysialylation Promotes Migration and Invasion

**Figure 5:**
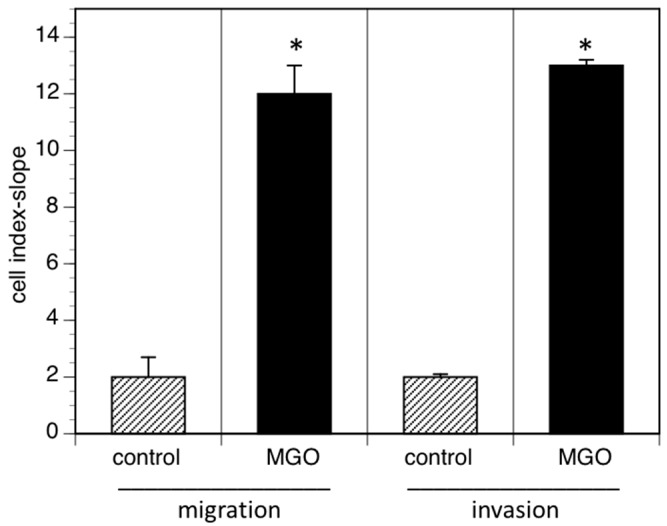
MGO-induced polysialylation promotes migration and invasion. A total of 0.5 × 10^6^ Kelly cells were grown in the absence (control) or presence of 100 µM MGO for 48 h prior to the migration or invasion assay. Migration/invasion in the absence of MGO was set to 100% (control) and in the presence of MGO in percent of the control. Bars represent means +/− SEM of 3 independent experiments (* *p* < 0.05).

Reduced cell adhesion correlates with cell migration or invasion into tissue and increased invasion of tumor cells often promotes metastasis. We therefore analyzed cell migration and invasion using the real time cell analyzer. Migration was measured using serum as chemoattractant, whereas invasion was quantified using EHS extra cellular matrix gels. When cells were grown in the presence of MGO, migration to the chemoattractant was dramatically increased (Figure 5). A similar effect was observed when the cellular invasion into EHS extra cellular matrix gels was analyzed (Figure 5).

## 4. Discussion

Methylglyoxal (MGO) is a regular byproduct of glycolysis [16], which is the major energy-generating pathway in the brain, since fatty acids cannot path the blood brain barrier [22,23]. Tumor cells are also known to use glucose as their primary energy source [14]. Therefore, many tumors such as neuroblastomas produce high amounts of MGO. MGO is a dicarbonyl-compound, and is known to be, in comparison to glucose, a very strong glycating agent [17].

In this study, we analyzed the effect of MGO on neuroblastoma cells and could demonstrate that neuroblastoma exhibited characteristics consistent with more aggressive disease, e.g., less adhesive and more invasive after glycation with MGO. One possible explanation for the reduced adhesion and enhanced migration could be the upregulation of polysialic acids after MGO treatment. Polysialic acid is nearly exclusively expressed on the neural cell adhesion molecule NCAM. Two polysialyltransferase (ST8Sia2 and ST8Sia4) are capable to polysialylate NCAM. Since we could demonstrate that NCAM expression is not altered after glycation e.g., treatment with MGO, we speculate that the increased expression of polysialic acid may be due to increased polysialyltransferase expression.

In this context it would be also important to know, how MGO or glycation alter the expression of polysialyltransferases. Although recent studies demonstrate that MGO alters gene expression profiles [24], the mechanism how MGO interfere with transcription is still unknown. It is conceivable that glycation of transcription factors is the major cause for this observation. In addition, it is also possible that polysialylation is only indirectly responsible for the reduced adhesion. The major adhesion molecules binding to ECM proteins such as laminin, vitronectin or fibronectin are integrins. It has been shown that glycation of ECM proteins interferes with integrin function [25,26], however whether the glycation of integrins itself interfere with its function is not known. However, not only adhesion, but also migration is affected by polysialylation. In this context, Li et al. (2011) proposed that polysialylation could act via an FGF-receptor dependent mechanism [27]. Further experiments including enzymatic removal of polysialic acid by Endo N after MGO treatment are necessary to finally answer these questions.

MGO is generated under physiological conditions in all cells including tumor cells. In our experiments we added additional MGO to the cell culture medium. The question arises: How physiological is this procedure? To answer this question, one has to keep in mind that under physiological conditions the oxygen concentrations are much lower than cell culture conditions, and therefore, the glycolysis rate is probably much higher than cell cultures. Therefore, it is well accepted to increase the MGO concentrations in cell culture to mimic the physiological conditions, in which such concentrations are reached.

Glycation of cells results in upregulation of RAGE and thereby stimulating inflammatory cascades in a positive feedback loop [28,29]. In this study we present data that RAGE expression is not changed after treatment of Kelly cells with 100 µM MGO. Therefore, we conclude that RAGE is not directly involved in neuroblastoma progression. However, there are studies suggesting an involvement of RAGE in tumor progression [30,31,32]. We have also performed FACS and Western blot experiments showing increased RAGE expression after MGO treatment (data not shown). However, we observed an increase in RAGE expression only after treatment with more than 300 µM MGO and these concentrations interfere with the viability of Kelly cells (see Figure 1).

Further arguments that glycation may promote tumor growth arises from data from diabetic patients. It is well known that both patients with diabetes type I and II have significant higher risks of developing cancer and have poorer prognosis [33,34]. As already mentioned, diabetes patients also have increased levels of MGO. Therefore, one could argue that at least the elevated levels of MGO are partially responsible on the molecular level for the increased tumor risk of diabetic patients.

Recently, we could demonstrate that glycation of immune cells has a negative impact on their function [35,36]. For example, NK-cells are less active to kill tumor cells after being glycated. This opens a further line of evidence that increase MGO levels due to diabetes or increased blood glucose promotes tumor progression.

In summary, we propose that glycation seems to be a negative event for tumor patients and increased production of MGO during tumor progression can alter its properties. Unfortunately, there is no treatment for “de-glycation” available at the moment there is a need to develop anti-glycation strategies in the future.

## 5. Conclusions

Methylglyoxal (MGO) is regular byproduct of glycolysis and occurs in high concentration in the brain. MGO is a dicarbonyl and highly reactive. It glycates proteins and advanced glycation end products are formed. In this study, we could demonstrate that glycation had several severe effects on neuroblastoma cells. It leads to increased expression of polysialic acid, a negative prognostic marker and interferes with adhesion and migration of neuroblastoma cells.
